# From NIL to real estate: strategic revenue adaptation in the post-house era of college athletics

**DOI:** 10.3389/fspor.2025.1728507

**Published:** 2026-01-15

**Authors:** Nikolas R. Webster, Jeffrey Carr

**Affiliations:** School of Kinesiology, University of Michigan, Ann Arbor, MI, United States

**Keywords:** athlete compensation, college athletics finance, *house* settlement, NIL, revenue sharing, sport-anchored real estate, strategy

## Abstract

Recent legal and financial reforms have altered the business model of college sports. The erosion of the NCAA's amateurism defense (culminating in the *House* settlement) has formalized athlete compensation and introduced direct labor expenses into athletic departments finances for the first time. Concurrently, the transfer mobility of athletes, new federal oversight, and the continuing evolution of NIL markets has increased competition among schools for athlete talent while also constricting institutional margins. With approximately 22% of athletics revenue designated for athlete compensation, universities now encounter a new financial mandate to either generate new revenue streams or accept new deficits with long-term fiscal consequences. Drawing from professional sports, this paper examines sport-anchored, mixed-use real estate development as a strategic mechanism for college athletic departments to generate new revenues for athlete labor costs. This conceptual analysis positions real estate development as a revenue hedge and strategic adaptation to the post-*House* financial environment.

## 1 Introduction

Over the last five years, the structure of college athletics has changed more dramatically than at any point since the formation of the NCAA. The combination of numerous antitrust rulings, the overall liberalization of NIL, and the *House* settlement has quickly dismantled the economic differences that long divided college and professional sport. NCAA universities are now responsible for compensating athletes who generate competitive and commercial value for their institutions. This introduction of direct athlete labor costs has created an immediate budgeting strain for athletic departments. Along with the rapidly growing name, image, and likeness (NIL) marketplace and an increasingly mobile athlete labor pool, this shift has significantly amplified the financial risks of roster construction and retention for college sport teams.

In response to this new environment, universities are actively searching for new, innovative, and sustainable sources of revenue. Recent reporting suggests that even the most resourced-advantaged conferences (e.g., SEC, Big Ten, etc.) are exploring external investment options, including private equity, as a means to stabilize future cash flow (1). This emerging pursuit of new, innovative financing signals a broader recognition that current revenue sources may be insufficient to meet the financial demands of the post-*House* era without compromising either competitive capability or institutional duty.

Against this backdrop, this paper examines one strategic pathway with precedent in the professional sport sector: sport-anchored mixed-use development. While scholars and practitioners have examined the legal and economic implications of NIL in college sport, little research has explored how institutions may adopt a professional sport real estate strategy as a sustainable, structural response to labor costs in the post-*House* era. For professional franchises, real estate has functioned not only as a supplementary business venture but as a financial hedge against escalating labor costs. Universities may pursue similar development strategies, using real estate as a financial adaptation and response to the financial reconfiguration of college sport.

This paper proceeds as follows. Section 2 reviews the transformation of finance and revenue generation within college athletics as well as the changes in the governance of college sport. Section 3 outlines the legal and regulatory evolution of athlete compensation, NIL, and financial implications post-*House*. Section 4 examines professional sport-anchored developments as a revenue diversification mechanism, provides examples from various professional leagues in North America, and documents early adoption of similar strategies by universities. Finally, Section 5 discusses the strategic implications of these developments for college athletic finance, competitive balance, and future research.

## 2 Finance and revenue generation in college athletics

The financial evolution of college athletics represents one of the most significant transformations within American higher education over the past century. What originated as an amateur-based operation has scaled into a multi-billion-dollar industry deeply embedded within complex legal, regulatory, and media ecosystems (2–4). This transformation has not only been driven by increases in consumer demand and technological advancements (e.g., television, streaming, etc.) but also by intentional institutional strategies to maximize visibility, prestige, and revenue through increasingly commercial and corporatized means. Although legally classified as 501(c) (3) nonprofits, university athletic departments act as revenue-generating businesses within a hyper-competitive, increasingly deregulated marketplace (2, 5, 6).

For much of the 20th century, college athletic departments relied on ticket sales and alumni donations as primary drivers of revenue, a consequence of the National Athletic Association's (NCAA) monopolistic control over broadcasting rights designed to preserve in-person attendance (3). The watershed moment came with the Supreme Court's decision in *NCAA v. Board of Regents of the University of Oklahoma* (1984) (7), which upended the NCAA's monopoly control over television rights, giving athletic conferences the ability to negotiate their own television rights, beginning the era of media-driven revenue generation. This shift away from centralized broadcast rights ownership (by the NCAA) to the conferences resulted in increases in media-related income for member schools, surpassing ticket sales as the primary financial engine for most Division 1 programs (8). As such, institutions (specifically, those in the emerging “Power 5^1^” conferences) began repositioning their athletic brands as monetizable assets that not only attracted fans, but also corporate partners, apparel companies, and media corporations.

During the 2000s and 2010s, R-1^2^ institutions' athletic departments began to resemble mid-size enterprises. For example, the University of Texas—Austin surpassed $200 million in annual athletic revenue prior to the COVID-19 pandemic, a figure that rivals the annual earnings of many professional sport teams (in 2024, the University of Texas—Austin reported revenues north of $330 million) (9). This revenue is derived from an increasingly diverse, entrepreneurial portfolio: media rights, NCAA tournament revenue distributions (“units”), ticket sales, royalties and licensing, donor contributions, apparel contracts, and corporate sponsorships. Specifically, institutions have followed media money, treating broadcast partnerships as primary strategic levers of their business model. Conference realignment (or more aptly, conference acquisition) decisions in the 2000s and 2010s were greatly influenced by media rights deals between conferences and broadcasting companies. Universities deliberately realigned conference affiliations to gain access to more lucrative television deals, highlighting the perception that conference membership is more about financial optimization rather than tradition and amateurism (10). As a result, broadcast contracts have risen into hundreds of millions of dollars, with the Big Ten and SEC conferences securing long-term deals that mirror those of professional sport leagues (see Table 1).

**Table 1:** Conferences, media partners, & contract valuations.

Conference	Total Value ($)	Average Annual Value ($)	Expiration Year	Media Partner(s)
Atlantic Coast Conference (ACC)	$4,800,000,000	$240,000,000	2036	ESPN
Big Ten Conference (B10)	$8,050,000,000	$1,150,000,000	2030	CBS/FOX/NBC
Big 12 Conference (B12)	$2,280,000,000	$380,000,000	2031	ESPN/FOX
Southeastern Conference (SEC)	$7,100,000,000	$710,000,000	2034	ESPN

Although the nonprofit status of institutions legally prohibits them from distributing profits, athletic departments deploy their revenues through *strategic* reinvestment. These reinvestments reflect calculated decisions to create competitive advantages in areas such as capital projects (stadium renovations, practice facilities, etc.), coaching salaries, athlete support services, and recruiting infrastructure. This conduct demonstrates the concept of *win maximization* (11, 12), where sport organizations (in this case, athletic departments) aim for a competitive advantage on the field as a pathway to financial sustainability and institutional viability (13). This reinvestment is not solely a budgeting choice, but rather an intentional, necessary decision to compete (on and off the field) within the competitive marketplace of college sport. This (along with the current fragmented landscape of college athletics) has resulted in a small number of institutions (primarily within the Power 4^3^ conferences) retaining higher levels of revenues compared to other colleges and universities that also participate in NCAA athletics.

In other words, this “winner-take-all” market creates an augmented level of stratification between institutions, where differences in television exposure, alumni base size, and donors drive the divide between the haves and have nots. For example, schools that can secure the commitments of top-ranked football recruits can translate those athletes into millions of dollars of athletic department revenue, demonstrating the intense competition for resource (labor, funding, etc.) acquisition (14).

Further, athletic departments have adopted managerial practices typical of private enterprise. Customer relationship management (CRM) systems have become customary within athletic departments to help engage with fans, students, alumni, and corporate partners. This shift reflects a broader recognition that revenue generation is not viewed as an auxiliary function, but rather as an essential strategic imperative that justifies expanded staffing, capital expenditures, and institutional political capital (15). The adoption of these business practices and the larger entrepreneurial behavior of college athletic department administrators is a result of the unstable environment that these organizations find themselves in.

This evolution of athletic departments into quasi-corporate entities has culminated to a crossroads moment: the longstanding model of indirect athlete “compensation” (via scholarships, training facilities, etc.) is no longer sufficient, legally defensible, or financially sustainable. Legal rulings and market forces have dismantled the amateurism framework that once provided the NCAA's regulatory authority (3, 16, 17), and now, college athletic departments must adapt to this changing landscape. As courts continue to confirm the legal and economic realities of college sport, institutions must reconcile their aggressive revenue generation models with the legal and financial reality of directly compensating athletes. In this new landscape, the question is no longer whether athletes should be paid, but rather, how will institutions fund, structure, and operationalize compensation models that also preserves their own competitive viability and institutional solvency.

## 3 Athlete compensation, name, image, & likeness (NIL), and the new financial reality

For over a century, the NCAA built its regulatory framework on the notion of amateurism (7): athletes playing college sport were *students* first, and therefore not able to receive compensation for their athletic contributions to member institutions. While schools were monetizing their athletic departments, they did not have direct labor costs responsible for producing the product itself (4). This model, however, is no longer viable, and athletes are not only able to profit from their name, image, and likeness (NIL), but also benefit from institutional revenue sharing. This shift represents not only a policy change, but a wholesale restructuring of the economic logic underpinning college sport (5).

The first splintering in the legal structure of amateurism appeared in *Board of Regents.* While the Supreme Court upheld the NCAA's governance role, the majority opinion described amateurism as central to college sport's commercial appeal. The dissenting opinion, however, argued that this justification masked anticompetitive behavior. The decision entrenched the amateurism classification of labor and granted the NCAA decades of protection from scrutiny (and decades of restricted compensation for athletes) (18). However, this shield weakened with *O'Bannon v. NCAA* (2014) (56). The Ninth Circuit ruled that prohibiting athletes from profiting from their NIL (particularly NIL in NCAA-branded EA Sports video games) violated antitrust law. *O'Bannon* exposed several contradictions in the NCAA's stance on amateurism (3), with the courts mentioning how tennis players could accept $10,000 in prize money before enrolling at NCAA member schools and that athletes could receive Pell Grants. *O'Bannon* did not produce direct pay-for-play, but it did strip amateurism of its antitrust defense and opened the door to broader challenges.

Less than a decade later, *NCAA v. Alston* (2021) (19) further weakened the NCAA's amateurism justification. In a unanimous decision, the Supreme Court found that the NCAA's restrictions on education-related benefits constituted unlawful price fixing. In the concurring opinion Justice Brett Kavanaugh wrote that “The NCAA's business model would be flatly illegal in almost any other industry in America…the NCAA is not above the law” (19). The *Alston* decision made clear that labor in collegiate athletics must be treated under the same antitrust principles as professional labor; that the market (not tradition) would determine one's value (6, 17).

The *House v. NCAA* settlement (reached in 2024 and approved in 2025), consolidated several lawsuits against the NCAA challenging restrictions on college athlete compensation. The agreement awarded $2.576 billion in damages to athletes denied NIL compensation^4^ as well as established a new revenue sharing system: schools may now allocate up to 22% of annual athletics revenue (about $20.5 million for a Power Four athletic department) to athlete compensation (20). Although athletes remain non-employees post-*House*, the settlement effectively established player compensation as an expense into athlete department accounting concerns for the first time (21). As a result of the settlement, athletic departments have been forced into budgetary compression; the fixed share of revenue earmarked for athletes significantly reduced flexibility for costs such as facilities, staff salaries, and non-revenue sports (22). In practice, compensation under *House* serves as a hard salary cap for schools as they face limits on direct compensation (i.e., revenue sharing). However, third party NIL markets create supplemental opportunities for athletes outside institutional control (like how professional athletes secure endorsement deals). While this “salary cap” model may help provide a competitive sporting environment between institutions, there is still a wide disparity between schools in regarding revenue generation, pushing universities toward more competitive financial innovation (16, 23) (see Table 2).

**Table 2:** Total revenues by conference (2023–2024) via knight-newhouse college athletics database.

Conference	Total Revenue (Billions)
Southeastern Conference	$2.592
Big Ten Conference	$2.360
Big 12 Conference	$1.555
Pacific-12 Conference	$1.341
Atlantic Coast Conference	$1.262
Mountain West Conference	$0.692
Sun Belt Conference	$0.517
American Athletic Conference	$0.513
Mid-American Conference	$0.435
Conference USA	$0.300

Soon after the *House* settlement, federal intervention reshaped the college sport NIL governance. On July 24, 2025, President Donald Trump issued the *Saving College Sports* Executive Order, declaring that pay-for-play arrangements are improper and should not be allowed by universities (24). To monitor third-party NIL deals post-*House,* the College Sports Commission (CSC) was created, a Deloitte-contracted entity charged with vetting NIL deals exceeding $600 via an online portal called *NIL Go*. The CSC reviews transactions for legitimate business purpose and market comparability and rejects agreements that appear either (1) performance-based or (2) not reflective of market-value rather than completely NIL-based (25). In its first *NIL Deal Flow Report* (26), the CSC documented 6,090 cleared deals totaling $35.4 million, averaging $5,816 per agreement (another 332 were rejected and 75 were resubmitted for inadequate documentation or invalid business purposes). Although the CSC's private-contractor status limits transparency (and it is not subject to FOIA^5^), its creation signals a new era of formal oversight (21). What was once an unregulated marketplace (post-*Alston*) now functions under a quasi-federal compliance structure intended to preserve competitive balance between schools while legitimizing NIL transactions (5, 21).

Since *House* was approved, the operational implications for universities have been profound. The *House* framework effectively transformed college athletic departments from primarily revenue-generating entities into a more professionalized model with new labor costs. To remain competitive in recruiting and retention, schools must now budget tens of millions annually for athlete compensation in addition to expanding operating costs. This has resulted in an acceleration towards professionalized athletic departments. Some examples include the University of Georgia's in-house NIL department (27), Stanford University appointing former NFL quarterback Andrew Luck as its *General Manager of Football* (28), and schools such as Coastal Carolina and Western Michigan have established general manager roles dedicated to NIL and roster management (29, 30). Unless athletic department revenues increase enough to offset the 22% revenue share allocation, athletic departments are likely to face long-term structural deficits (10).

In the landscape of college athletics, the sustainability of the enterprise will depend on innovation beyond existing and traditional revenue streams. Universities compete in multiple markets (talent acquisition, sponsorship, fan engagement, etc.) and must adopt a strategic approach to remain viable (12). The capacity to (1) balance compliance with entrepreneurial responsiveness, (2) diversify income streams, and (3) align athletic operations with community (e.g., fans, alumni, etc.) objectives will define the next decade of college sport (31, 32). NIL did not end completely end amateurism; it marked the beginning of a managerial era in which the success of collegiate athletics depends on strategic foresight and structural adaptation by college athletic departments.

## 4 Insights from professional sports: entrepreneurial revenue generation & mixed-use real estate investments

**Figure 1:**
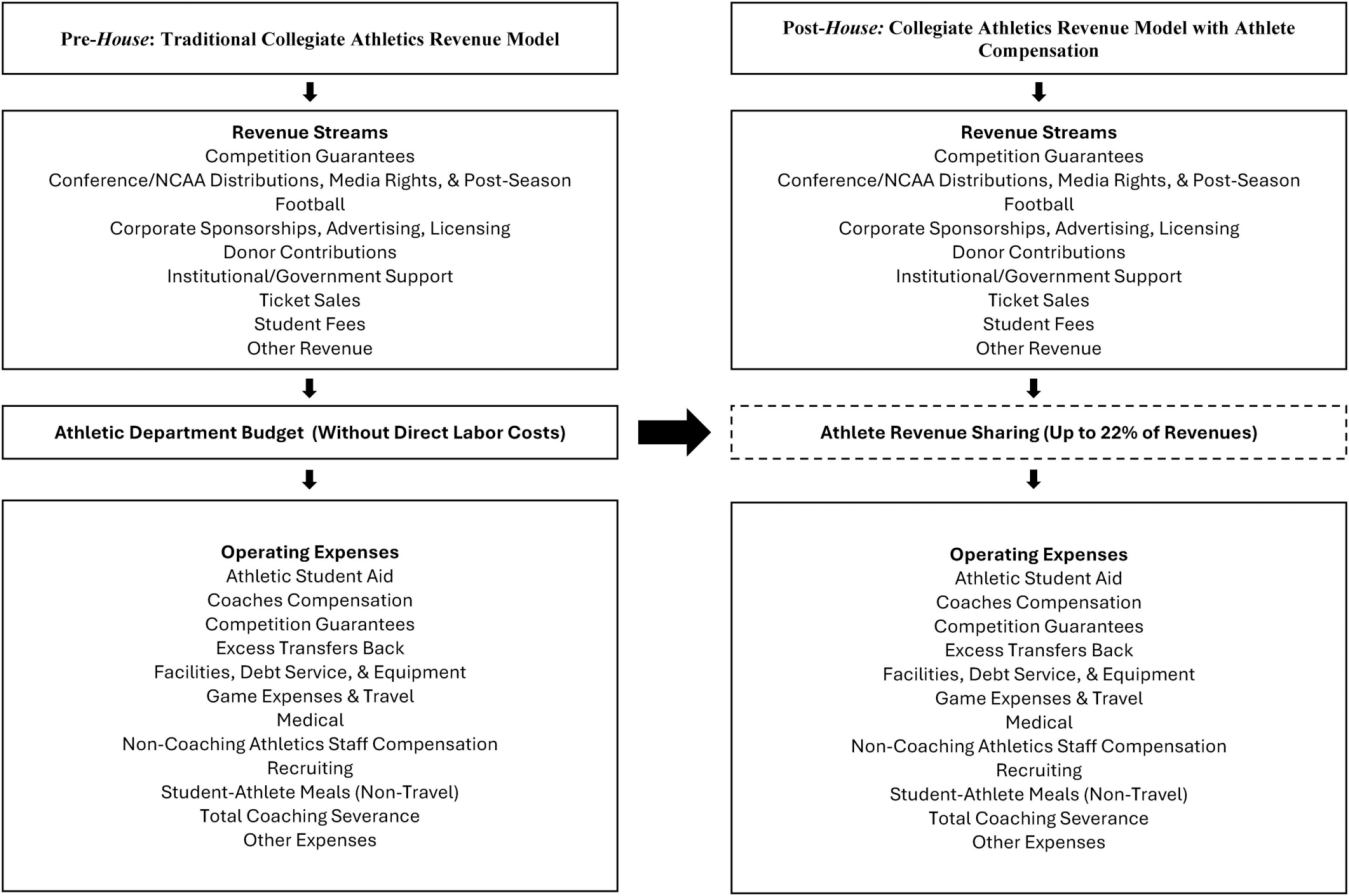
Conceptual model of collegiate athletics revenue channels before & after the *house* settlement (revenue and expense categories reflect standardized NCAA division I financial reporting classifications as defined by the knight-newhouse college athletics database).

Though it took nearly 40 years from *Board of Regents* to *House* and the subsequent creation of *NIL Go*, the outcomes of this legal process have immediate fiscal implications for NCAA member institutions. For the first time in NCAA history, schools can directly fund their athletic labor. This new expense is not theoretical. In the current free-market transfer era, schools must spend their full allotment of this revenue sharing “salary cap” to remain competitive in recruiting and retention of athletes. If a Power 4 school chooses to not engage, their competitors almost certainly will. Figure 1 demonstrates this shift by illustrating how athlete compensation alters the internal flow of revenues and expenses within athletic departments relative to the pre-*House* model.

From a traditional economic profit maximization perspective in professional sports, teams aim to compensate players based on their *marginal revenue product* (MRP; 35). In other words, ownership is willing to compensate a player up to the amount of additional revenue they bring the franchise. In the collegiate context, however, the profit maximization assumption is complicated by universities' nonprofit status and educational mission (despite the multi-billion-dollar endowments many Division-I schools have accrued). Whether viewed through a win maximization paradigm (11, 12) or a profit maximization perspective (33), the practical result is the same for college athletic departments: athletic labor now carries an explicit cost. And for decision-makers (i.e., athletic directors and school administration), this additional expense stimulates the need to explore new, untapped revenue streams (1).

Professional teams have long mitigated labor costs by diversifying into additional revenue streams. These revenue streams have included in-venue food and beverage sales, signage, venue naming rights, media (e.g., television, radio, streaming, etc.), licensing, and many others (34). Many of these are classified as “sport-derived revenues” as the athletes are responsible for the generation of these revenue streams. Historically, labor in the professional leagues have been able to secure a share of these revenues via collective bargaining agreements (CBAs). In *House*, a specific percentage of athletic department revenues were used in calculation of the “salary cap”, though these specific revenue streams were not explicitly named (20). In professional league CBAs, however, stadium-adjacent revenues (i.e., real estate development) are excluded from the shareable pool of revenues that are used for player salaries.

The National Football League's (NFL) CBA, for instance, specifies that revenues from real estate development (in conjunction with or related to any stadium lease) are not counted towards sport-derived revenues used for salary cap calculations (35). Related, the National Hockey League's (NHL) definition of “hockey-related revenue” excludes revenues from the sale or leasing of real estate (36). Similar language appears in Major League Baseball (MLB) and the National Basketball Association's (NBA) CBA, where the owners retain substantial income streams from property holdings beyond the reach of revenue sharing established by CBA. This notion reflects a larger reality of professional sport leagues where franchises generate enormous spatial concentration of fans and leisure spending. In the late 20th century, many professional team owners, seeking new revenue streams, understood that getting fans on their venue's footprint for additional time before and after games, and even on non-gamedays, was an effective way to leverage the team to create additional revenue streams. As a result, team owners began leveraging stadium, venue, and ballpark attendance into broader, year-round economic ecosystems: using real estate investment as a strategy (37, 38) to redefine the business model of sport. Specifically, owners have developed sport-anchored mixed-use districts as an innovative, sustainable revenue stream.

The origin of sport-anchored (i.e., mixed-use entertainment) districts can be traced back to Indianapolis's downtown redevelopment in the 1980s (39), and perhaps even earlier to Las Vegas's emergence as an entertainment hub. However, the construction of San Diego's Petco Park and Ballpark District represented a substantial turning point within professional sport and real estate development. Following voter approval of Proposition C in 1998 (40), Padres owner John Moores agreed to finance nearly $500 million in adjacent mixed-use development, transforming a blighted district into a vibrant urban neighborhood. By 2016, the district's assessed value had grown to at least $2.87 billion, with some estimates exceeding $13 billion (41, 42). The project demonstrated how ballpark-anchored real estate could produce sustained returns far beyond traditional sport-related revenue streams (ticket sales, concessions, etc.).

Subsequent projects such as L.A. Live in Los Angeles, Titletown in Green Bay, and The Battery in Atlanta replicated this mixed-use development model at different scales. L.A. Live, a $2.5 billion entertainment district surrounding Crypto.com arena, draws more than twenty million visitors annually and integrates retail, hospitality, and residential assets within the event footprint (43, 44). Though many professional sport franchises (NBA's Lakers, WNBA's Sparks, & NHL's Kings) as well as shows and concerts provide high density foot traffic on numerous event days in downtown Los Angeles, L.A. Live expands upon the entertainment footprint to create a synergistic district.

Titletown (adjacent to Lambeau Field in Green Bay, Wisconsin) applied the same logic in a much smaller market. The mixed-use district, anchored by Lambeau Field and other participatory sport offerings (e.g., public football field, playground, ice skating rink, etc.), was successful in securing a partnership agreement with Microsoft to create *Titletown Tech*. Titletown serves as an example that the concept of sport-anchored developments can be successful in non-major markets (e.g., Green Bay) if a strong anchor asset exists (in this instance, Lambeau Field). The Battery, home to the Atlanta Braves of MLB, surrounds Truist Park, and epitomizes the integration of franchise-owned real estate. The Braves' ownership group maintains direct control of the surrounding properties that include a variety of restaurant and entertainment assets in additional to residential and retail spaces (45). Like Moores did in San Diego, these examples (Titletown and The Battery) illustrate a shift in ownership behavior from generating revenue solely through sport performance (i.e., the team) to creating place-based economic generators that enhance and sustain cash flow while hedging against player compensation volatility (changes to CBAs, salary cap fluctuations, etc.).

For universities, the mixed-use development model from professional leagues offers both precedent and opportunity. As collegiate athletics increasingly mirrors professional sport regarding its financial structure, it is logical for schools to look towards real estate development as a strategic response to rising labor costs. In fact, some schools have even begun to engage in this practice themselves. For example, Michigan State University issued a request for proposals (RFP) in 2024 for a 14-acre mixed-use development featuring housing, retail, and a hotel, all anchored by a new Olympic sports arena (46). The project employes a ground-lease structure that allows private developers to operate the site while the university retains land ownership (a model that follows professional franchise real estate portfolios).

Another example is Iowa State University, who broke ground in 2025 on CyTown, one of the first multi-use districts built on a college campus. Bookended by Iowa State's football stadium and basketball arena, CyTown's assets will include a health clinic, a non-student housing suite product (deemed “CyTown Suites”), food, retail, and office spaces, and an amphitheater (47). Similarly, the University of South Carolina issued an RFP in 2024 to develop around the school's football stadium. With over 800 acres of developable land along the Congaree River, the university aims for its football facilities serving as anchor assets for the project (48). Other examples of schools exploring sport-anchored development districts include the Gateway District for The University of Kansas and the mixed-use Neyland Entertainment District for the University of Tennessee (49, 50).

These examples come from Power Four conference schools, specifically, institutions that are now engaging in the new post-*House* revenue sharing. As schools from these conferences have historically been more financially successful at the top level of Division-I sports (9), it is likely that these schools will be more incentivized to use a larger share of their “salary caps” established by *House* in recruiting and talent retention. *House* itself acknowledged this fact, that schools in the Group of Five^6^ did not generate as much revenue as Power Five^7^ schools and were likely to spend less than they were permitted to spend on student-athlete compensation (though they implemented the same shared-revenue pool ceiling for all schools) (19). Additionally, Power Four conference schools are uniquely positioned as opposed to some of their mid major counterparts (e.g., MAC, Mountain West, Sun Belt, etc.) in terms of their institutional brand equity (51).

Regardless of conference affiliation, each university must report its athletic finances to the NCAA's Member Financial Reporting System (MFRS). These reports do not list sport-related real estate development as a revenue stream for universities (9). The absence of a standardized line item in MFRS records suggests that university land holdings are managed outside of athletic departments and instead are captured on each school's overall financial statements.

There are various mechanisms by which a university could strategically navigate sport-anchored real estate development. While not an exhaustive list, a university may choose to (1) sell land to a private developer, (2) enter a ground lease with a private developer, or (3) partner with a private developer while maintaining ownership of university land. For example, in estimating a hypothetical valuation for a sale of land sufficient to offset the post-*House* $20.5 million annual revenue share, land may need to be valued between $500 million to over a billion dollars (contingent on discount rate and contractual agreements; see Table 3).

**Table 3:** Hypothetical land sale valuation to cover annual revenue share.

Initial Revenue Needed (3% Annual Growth)	Discount Rate	Land Valuation
$20.5 million (100% of revenue share covered)	5%	$1.025 billion
$20.5 million (100% of revenue share covered)	6%	$683 million
$10.25 million (50% of revenue share covered)	5%	$513 million
$10.25 million (50% of revenue share covered)	6%	$342 million

If developer partnerships are utilized, one should consider recent RFPs from University of Texas—Austin and the University of Mississippi (Ole Miss). For example, University of Texas—Austin is currently soliciting a private developer to build housing for 300 student athletes at an annual rental payment of $4.32 million (escalating by 3% annually) (52). Concurrently, Ole Miss seeks to develop a sport-anchored district containing 130 hotel and condominium units and 35,000 square feet of retail and dining spaces (53). While the scale of university ventures may differ, the overall strategy is intended to create sustainable, long-term revenue streams that aid the university's finances in a post-*House* revenue sharing era.

Professional league CBAs provide precedent that sport-anchored real estate-related revenues are not subject to revenue sharing. At this time, college athletes have not formally and comprehensively organized as a labor union or have collectively bargained. However, scholars have suggested that collective bargaining within college athletics could not only be a solution to reoccurring antitrust ligation, but also a mechanism to formalize revenue sharing agreements (21). Questions remain about the accounting pathways schools must use to direct revenue streams from sport-anchored developments to sustainably fund their revenue sharing model. Until greater clarity emerges over which revenues are subject to revenue sharing, sport-anchored real estate remains one of the most plausible, sustainable, and scalable strategies for universities to relieve the newfound pressure of compensating labor.

## 5 Discussion

Following decades of legal decisions and structural shifts eroding the NCAA's positioning of “amateurism”, culminating with the approval of the *House* settlement, the collegiate sport landscape has fully transitioned into what many would consider a professional-style model (including a professional labor market). College athletes may now be compensated by the institution for which they play while simultaneously participate in sponsorship (NIL) agreements. Further, the nonprofit structure of college and universities places onus on win maximization rather than profit maximization. Athletic departments now face a clear financial problem where they must offset this new expense while also increasing revenue. If not, they face either a revenue deficit with potential negative long-term consequences or a competitive disadvantage within a win maximization environment. In either scenario, the new paradigm is one where revenue generation is strategically inseparable from competitive viability.

Mixed-use districts built around sport facilities have already proven to produce recurring revenue that does not depend on competition or ticket sales. Across markets (varying in size and demographic composition), professional franchises have successfully leveraged sport and event attendance to capture adjacent spending through mixed-use districts. These precedents can serve as a strategic, guiding direction for college athletic departments as a structural hedge against rising labor costs. In the post-*House* environment, athletic administrators need to act innovatively regarding revenue generation, and the mixed-used model provides that innovation.

Despite industry success of mixed-use districts regarding revenue generation, there is literature discussing the economic limitations of sport venues and sport districts (45). Notably, this body of literature concludes that any localized economic impacts are generally minimal in scale and only positively benefit closely related economic sectors (54, 55). The present manuscript does not seek to challenge that consensus, but rather, looks to expand upon the scope of that research. If one concedes that any collegiate development does not (or will not) generate new economic activity and only relocates it, that result could be considered a positive outcome for universities. If universities are now required to pay labor, then relocating localized spending (e.g., retail, residential, entertainment) onto their university footprint may be strategically beneficial, akin to municipal competition for tax revenues (54). However, any market-level welfare gains must be considered against the potential negative externalities for other local businesses and developers (55).

Future research should investigate whether collegiate sport-anchored developments behave economically (and politically) like their professional counterparts or whether nonprofit governance, land ownership structures, and mission-based constraints produce different outcomes. If the post-*House* era induces a wave of university-led development tied to labor cost pressures, this may represent not only a structural transformation in athletic finance but the emergence of a new branch of literature at the intersection of antitrust law, higher education finance, and sport-based development strategy.

## Footnotes

^1^The “Power 5” refers to the five conferences historically considered to have the greatest media exposure and financial resources in Division I athletics (Atlantic Coast Conference[ACC], Big Ten Conference [Big Ten], Big 12 Conference [Big 12], Pac-12 Conference [Pac-12], and the Southeastern Conference [SEC]).

^2^R-1 refers to the Carnegie Classification of Institutions of Higher Education, denoting doctoral universities with very high research activity. This is the highest tier of research designation in the Carnegie system.

^3^Following recent conference realignment in which most Pac-12 institutions departed for other conferences, the term “Power 4” is now commonly used to describe the ACC, Big Ten, Big 12, and SEC.

^4^Damages were awarded to athletes who competed from the 2016/2017 to 2023/2024 academic years as well as injunction relief for current players.

^5^The Freedom of Information Act (FOIA) is a U.S. federal law that grants the public the right to access records from government agencies, subject to certain exemptions for privacy, national security, and other protected interests.

^6^The “Group of Five” refers to the conferences (American, Conference USA [CUSA], Mid-American Conference [MAC], Mountain West, Sun Belt) that do not have the media exposure and financial resources of Power 4 institutions. Given realignment within the Pac-12 conference, the Pac-12 has recently been considered a Group of Five (or Group of Six) conference.

^7^As *House* was being negotiated the Pacific 12 (Pac-12) conference was still in existence and as such referenced the Power Five instead of the Power Four.
